# The Terrestrial Isopod Microbiome: An All-in-One Toolbox for Animal–Microbe Interactions of Ecological Relevance

**DOI:** 10.3389/fmicb.2016.01472

**Published:** 2016-09-23

**Authors:** Didier Bouchon, Martin Zimmer, Jessica Dittmer

**Affiliations:** ^1^UMR CNRS 7267, Ecologie et Biologie des Interactions, Université de PoitiersPoitiers, France; ^2^Leibniz Center for Tropical Marine EcologyBremen, Germany; ^3^Rowland Institute at Harvard, Harvard University, CambridgeMA, USA

**Keywords:** bacterial diversity, endosymbionts, microbiota, symbiosis, ecological interactions, terrestrial isopods

## Abstract

Bacterial symbionts represent essential drivers of arthropod ecology and evolution, influencing host traits such as nutrition, reproduction, immunity, and speciation. However, the majority of work on arthropod microbiota has been conducted in insects and more studies in non-model species across different ecological niches will be needed to complete our understanding of host–microbiota interactions. In this review, we present terrestrial isopod crustaceans as an emerging model organism to investigate symbiotic associations with potential relevance to ecosystem functioning. Terrestrial isopods comprise a group of crustaceans that have evolved a terrestrial lifestyle and represent keystone species in terrestrial ecosystems, contributing to the decomposition of organic matter and regulating the microbial food web. Since their nutrition is based on plant detritus, it has long been suspected that bacterial symbionts located in the digestive tissues might play an important role in host nutrition via the provisioning of digestive enzymes, thereby enabling the utilization of recalcitrant food compounds (e.g., cellulose or lignins). If this were the case, then (i) the acquisition of these bacteria might have been an important evolutionary prerequisite for the colonization of land by isopods, and (ii) these bacterial symbionts would directly mediate the role of their hosts in ecosystem functioning. Several bacterial symbionts have indeed been discovered in the midgut caeca of terrestrial isopods and some of them might be specific to this group of animals (i.e., *Candidatus* Hepatoplasma crinochetorum, *Candidatus* Hepatincola porcellionum, and *Rhabdochlamydia porcellionis*), while others are well-known intracellular pathogens (*Rickettsiella* spp.) or reproductive parasites (*Wolbachia* sp.). Moreover, a recent investigation of the microbiota in *Armadillidium vulgare* has revealed that this species harbors a highly diverse bacterial community which varies between host populations, suggesting an important share of environmental microbes in the host-associated microbiota. In this review, we synthesize our current knowledge on the terrestrial isopod microbiome and identify future directions to (i) fully understand the functional roles of particular bacteria (both intracellular or intestinal symbionts and environmental gut passengers), and (ii) whether and how the host-associated microbiota could influence the performance of terrestrial isopods as keystone species in soil ecosystems.

## Introduction

Over the last decade, research on animal–bacterial symbioses has shifted from its historical focus on binary host–symbiont interactions to a more holistic perspective, taking into account that symbioses are shaped by complex multipartite interactions between hosts and diverse symbiotic communities (the microbiome), partly acquired from the environment. This “microbiome revolution” (Blaser, 2014) was mainly driven by the advent of new high-throughput DNA sequencing technologies, which enabled us to appreciate the diversity and ubiquity of animal-associated microbiomes as an essential component of host biology as well as a source of evolutionary novelty (Ley et al., 2008; Fraune and Bosch, 2010; McFall-Ngai et al., 2013). Hence, it is now established that the microbiome can modulate host development (Shin et al., 2011), nutrition (Chandler et al., 2011; He et al., 2013; Wong et al., 2014), immunity (Chu and Mazmanian, 2013), vector competence and susceptibility to pathogen infection (Dong et al., 2009; Koch and Schmid-Hempel, 2012), and speciation (Brucker and Bordenstein, 2012, 2013).

The majority of studies on arthropod microbiomes have been conducted in insects and have made enormous contributions to our understanding of intricate host–symbiont relationships. For instance, many insects feeding on nutrient-deficient diets such as plant sap or vertebrate blood maintain long-lasting associations with heritable obligate mutualistic endosymbionts (primary symbionts) which provide essential nutrients (amino acids and vitamins) lacking from the host’s diet (Thao and Baumann, 2004; Moran et al., 2005; Pais et al., 2008; McCutcheon et al., 2009; McCutcheon and Moran, 2010). These symbionts are usually harbored in specialized cells (the bacteriocytes), which may form an additional host organ, the bacteriome. An even higher number of species harbor facultative intracellular symbionts (secondary symbionts). These are generally not required for host survival and reproduction, but may be beneficial under certain environmental conditions, for instance through increased thermal tolerance, host plant speciation, predator avoidance and defense against natural enemies and parasites (Montllor et al., 2002; Oliver et al., 2003, 2005; Ferrari et al., 2004; Tsuchida et al., 2004, 2010; Scarborough et al., 2005; Jaenike et al., 2010; Lukasik et al., 2013). Other facultative bacteria act as reproductive parasites and manipulate host reproduction in order to promote their own vertical transmission from mother to offspring (Duron et al., 2008; Hurst and Frost, 2015). The most common phenotype is cytoplasmic incompatibility (CI), a reproductive incompatibility between sperm and egg preventing normal mitosis (Tram and Sullivan, 2002; Serbus et al., 2008). Other reproductive manipulations result in female-biased sex-ratios that increase the number of infected females in host populations (via parthenogenesis, male-killing, or the feminization of genetic males), thereby enhancing maternal symbiont transmission (Stouthamer et al., 1993; Hurst et al., 2003; Narita et al., 2007; Bouchon et al., 2008). Bacteria of the genus *Wolbachia* are by far the most frequently encountered reproductive parasites and manifest the widest spectrum of reproductive manipulations (Hilgenboecker et al., 2008; Werren et al., 2008; Sicard et al., 2014b).

When considering the wider microbial community beyond heritable endosymbionts, many insect microbiotas are of relatively low diversity but highly specialized, such as in bees and flies (Koch and Schmid-Hempel, 2011; Martinson et al., 2011; Wong et al., 2011). At the other end of the spectrum are the termites with their highly diverse hindgut community of bacteria, archaea, and protozoans involved in lignocellulose digestion (Hongoh, 2010; He et al., 2013; Peterson et al., 2015). However, only very few studies have investigated microbiome patterns across ecologically diverse groups of insects, with the exception of Colman et al. (2012). Their study demonstrated that microbiota composition was strongly influenced by host diet in various insects. Specifically, bacterial diversity was highest in detritivorous and xylophagous species from different insect orders (termites and beetles) and their microbiotas were more similar to each other compared to insects with other diets. Overall, however, more studies in diverse non-model arthropods across different ecological niches will be needed to complete our understanding of host–microbiota interactions beyond the insects. In this review, we present terrestrial isopod crustaceans as an emerging model organism to investigate arthropod–bacteria symbioses of evolutionary and ecological relevance.

## Terrestrial Isopod Ecology and Evolution

**FIGURE 1:**
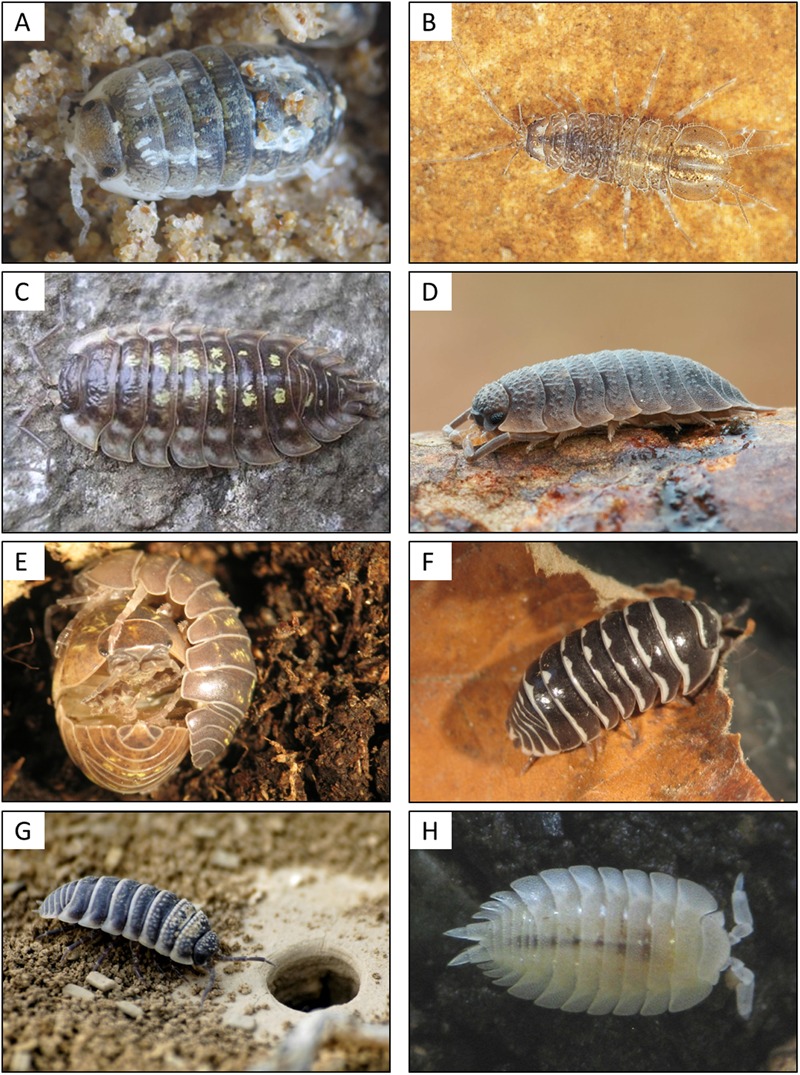
**Diversity of terrestrial isopods from littoral to desert habitats. (A)** The semi-terrestrial *Tylos europaeus*, inhabiting the littoral zone; **(B)** the freshwater isopod *Asellus aquaticus*; **(C)** the common woodlouse *Oniscus asellus*; **(D)** the rough woodlouse *Porcellio scaber*; **(E)** the pillbug *Armadillidum vulgare* during mating; **(F)** the “zebra pillbug” *Armadillidium maculatum*; **(G)** the desert isopod *Hemilepistus reaumuri* near the entrance of a burrow in Tunisia; and **(H)** the ant woodlouse *Platyarthrus hoffmannseggii*. This species is blind and lives in ant nests. Picture credit: Martin Zimmer **(B)**, Sören Franzenburg **(D)**, Giuseppe Montesanto **(G)**, UMR CNRS 7267 **(A,C,E,F,H)**.

From an evolutionary perspective, oniscidean isopods are the most successful colonizers of terrestrial habitats among the Crustacea, comprising around 3700 species (Schmalfuss, 2003). Originating from the marine littoral zone, they have colonized almost all types of terrestrial environments on Earth, including deserts, except for high alpine and polar regions (**Figure 1**). This makes them excellent model organisms regarding evolutionary adaptations to a terrestrial lifestyle in terms of reproduction, respiration, nutrition, excretion systems, and protection against desiccation (reviewed in Hornung, 2011). The main morphological and physiological changes compared to marine crustaceans include (i) a reduction in body size, (ii) a water-resistant cuticle, (iii) pleopodal lungs, (iv) a water conducting system, and (v) development of juveniles in a closed brood pouch (marsupium; reviewed in Hornung, 2011). In addition, they had to adapt to the food sources available in terrestrial environments (reviewed in Zimmer, 2002). Extant terrestrial isopods are detritivores, i.e., they feed on dead and decaying organic matter. Therefore, they represent a keystone species for plant litter decomposition in terrestrial ecosystems, contribute to nutrient cycling and regulate soil microbial activity (Zimmer and Topp, 1999; Zimmer, 2002; Crowther et al., 2015).

While plant material represents one of the most abundant sources of fixed carbon and nitrogen on the planet, it mainly consists of complex lignocellulose polymers that hinder the digestive utilization of this carbon and nitrogen. Obtaining nutrients from these compounds requires a complex set of enzymes allowing the degradation of lignins as well as cellulose. Historically, it has long been believed that the majority of animals do not possess the necessary enzymatic machinery and instead rely on symbiotic microorganisms (e.g., ruminants or termites). However, this view is increasingly challenged by the detection of endogenous cellulases in various animals (reviewed in Watanabe et al., 1998; Davison and Blaxter, 2005; Cragg et al., 2015), including isopod crustaceans (King et al., 2010; Kostanjsek et al., 2010; Kern et al., 2013). Nonetheless, most animal species studied to date still rely on symbiont-derived enzymes for cellulose degradation, either alone or in combination with host-derived endogenous enzymes (Cragg et al., 2015). Therefore, the role of bacteria (both environmental bacteria and symbionts located in the digestive tissues) in terrestrial isopod nutrition and fitness has attracted considerable attention in the last decades. First, isopods are constantly in contact with environmental microbes in the soil or ingested with their plant food sources and/or via feeding on their congeners’ feces (coprophagy; Gunnarsson and Tunlid, 1986; Zimmer and Topp, 2000; Zimmer et al., 2003; Ihnen and Zimmer, 2008). Second, it has long been suspected that bacterial symbionts located in the digestive tissues might play an important role in host nutrition via the provisioning of digestive enzymes (Zimmer and Topp, 1998a,b; Zimmer et al., 2001, 2002; Zimmer and Bartholmé, 2003). If this were the case, then (i) the acquisition of these bacteria might have been an important evolutionary prerequisite for the colonization of land by isopods, and (ii) these bacterial symbionts would directly mediate how their hosts contribute to important ecosystem processes such as decomposition and nutrient cycling. Several bacterial symbionts have indeed been discovered in the midgut caeca and the hindgut of terrestrial isopods and some of them might be specific to this group of animals (i.e., *Candidatus* Hepatoplasma crinochetorum, *Candidatus* Hepatincola porcellionum, and *Rhabdochlamydia porcellionis*; Kostanjsek et al., 2004b, 2007; Wang et al., 2004a,b), while others are well-known intracellular pathogens (*Rickettsiella* spp.) or reproductive parasites (*Wolbachia* sp.) infecting various insects as well (Werren et al., 2008; Bouchon et al., 2011; Leclerque and Kleespies, 2012; Kleespies et al., 2014; Sicard et al., 2014b). Moreover, a recent investigation of the microbiota in *Armadillidium vulgare* has revealed a high bacterial density and diversity in various tissues (Dittmer et al., 2014, 2016). In this review, we synthesize our current knowledge regarding the functional roles of particular bacteria in the terrestrial isopod microbiome (both intracellular or intestinal symbionts and environmental bacteria) and discuss to what extent this microbiome could (i) influence the performance of terrestrial isopods as keystone species of the soil macrofauna and (ii) represent a potential source of adaptive capacity in changing environments.

## A Diverse Symbiont Assembly

As for many symbioses, the association between isopods and microorganisms has initially been viewed as highly specific binary interactions between one host and one symbiont. We present the current knowledge of bacterial symbionts described in terrestrial isopods. Most of these bacteria where first discovered through microscopic observations and/or through ecological traits and all of them are intracellular or extracellular symbionts that are facultative for the host. Nicely illustrating the initial concept of symbiosis introduced by de Bary (1879), they represent a large scope of host–symbiont associations, including reproductive parasites (*Wolbachia*), bacterial pathogens (*Rickettsiella* and *Rhabdochlamydia*) and several symbionts specifically associated with digestive tissues, namely *Candidatus* Hepatincola, *Candidatus* Hepatoplasma, and *Candidatus* Bacilloplasma. Although most research has been dedicated to bacterial symbionts of terrestrial isopods, we expand this section to include viruses and acanthocephalan parasites known to infect this group of animals.

### *Wolbachia*, A Model to Study Genetic Conflicts

**FIGURE 2:**
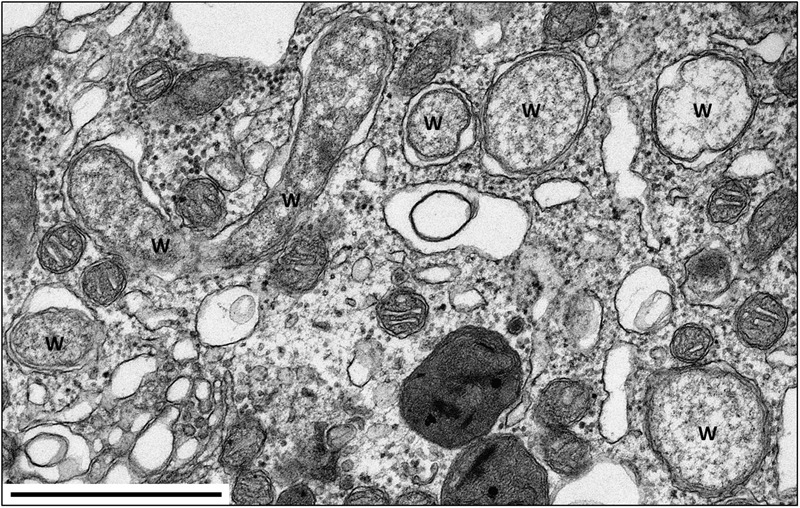
**TEM of *Wolbachia* in the nerve cord of *A. vulgare*.** W = *Wolbachia*. Scale bar: 1 μm. Picture credit: Maryline Raimond.

*Wolbachia pipientis* (hereafter *Wolbachia*, **Figure 2**) are obligate intracellular Alphaproteobacteria (order Rickettsiales) that are widespread in arthropods and also found in nematodes (Werren et al., 2008). Their capability to manipulate host reproduction has received considerable attention and has established them as reproductive parasites. Presence of *Wolbachia* in isopods was first observed in the pill bug *A. vulgare* as *Rickettsia-*like bacteria (Martin et al., 1973) that were later formally assigned to *Wolbachia* (Rigaud et al., 1991; Rousset et al., 1992). Terrestrial isopods have long been known to be prone to reproductive alterations such as intersexuality, sex ratio distortions or incompatible crosses (Legrand et al., 1987). In this context, identifying *Wolbachia* in *A. vulgare* emerged from a long series of studies looking for both physiological and genetic factors responsible for sex ratio distortions (reviewed in Bouchon et al., 2008).

The most extensive investigations have been performed on *A. vulgare* where, as observed in numerous terrestrial isopods, unisexual progenies are frequent. Taking advantage of the fact that isopods are highly tractable, especially for grafting experiments, sex reversals can be easily achieved through experimental tissue implants, indicating that sex determination and sex differentiation are very labile. Such experiments were used to demonstrate that *A. vulgare* females are heterogametic (WZ) and that unusual genetic combinations (WW or ZZ) are viable and fertile (Legrand et al., 1987). The corpus of these investigations led to propose an integrative model of the evolution of sex determination where *Wolbachia* plays a central role (Rigaud et al., 1997; Cordaux et al., 2011).

To date, three *Wolbachia* strains (*w*VulC, *w*VulM, and *w*VulP) have been identified in the pill bug (Cordaux et al., 2004; Verne et al., 2007). These *Wolbachia* are generally present in all tissues including gonads, hemocytes, nervous and digestive tissues (Dittmer et al., 2014; **Figures 2** and **5**). Interestingly, quantitative investigations also showed that the within-host *Wolbachia* distribution depends on both the *Wolbachia* strain and the tissue type, but not on host genotype (Dittmer et al., 2014). While it is expected that *Wolbachia* are most abundant in the ovaries, the target tissue for transgenerational transmission of obligate endosymbionts, the systemic infection of *Wolbachia* in terrestrial isopods could also play a role in the association (Sicard et al., 2014b): when transmitted from mothers to offspring, *Wolbachia* reverse male embryos into functional females, sometimes leading to all-female progenies. This remarkable aptitude to infect offspring may rely on a re-infection of ovaries from somatic tissues instead of direct cellular segregation between oogonia and oocytes (Genty et al., 2014). This manipulation called feminization is a *Wolbachia* strategy (or extended phenotype) to optimize their vertical transmission. As feminized ZZ males produce females without transmitting any W chromosome, the outcome is the rapid elimination of the W chromosome from infected populations. Therefore, in a population where all individuals are homogametic ZZ, the sex determination is under the control of *Wolbachia*. Hence, the *A. vulgare*/*Wolbachia* interaction is a perfect example of cytoplasmic sex determination (Rigaud et al., 1997; Cordaux et al., 2011). The precise molecular mechanism of feminization in *A. vulgare* still remains unknown but is achieved by preventing androgenic gland differentiation, the tissue where the androgenic (masculinizing) hormone (AH) is synthetized (Grève et al., 2004; Cerveau et al., 2014). The key role of the AH has been demonstrated by means of androgenic gland implants and injections of AH extract into young females, which were then reversed into functional males. When doing the same experiments with young females infected with *Wolbachia*, no sex reversal was recorded. Therefore, feminization could result from inhibition of androgenic gland development by targeting the hormonal sex differentiation in *A. vulgare*.

Apart from terrestrial isopods, *Wolbachia*-mediated feminization has only been observed in three insects: in *Eurema* butterflies (Hiroki et al., 2002; Narita et al., 2007; Kern et al., 2015), the leafhopper *Zyginidia pullula* (Negri et al., 2006, 2009) and the moth *Ostrinia scapulalis* (Sugimoto and Ishikawa, 2012), although the latter case is more complex in that *Wolbachia* achieves male-killing through a feminizing factor which induces the female splice variant of the sex determination gene *doublesex*. This results in a lethal mismatch between genetic and phenotypic sex determination in male embryos (Sugimoto and Ishikawa, 2012). In *Eurema* butterflies, on the other hand, *Wolbachia* acts at a later stage and achieves feminization during larval development through an as yet unknown mechanism (Narita et al., 2007), whereas *Wolbachia* reverses the sex-specific genome methylation pattern in *Z. pullula* males (Negri et al., 2009). Together, these observations in insects and terrestrial isopods illustrate *Wolbachia*’s ability to achieve the same phenotype in hosts with diverse sex determination systems.

Feminization is not the only *Wolbachia*-mediated manipulation observed in terrestrial isopods. To date, CI, a post-zygotic reproductive isolation frequently induced by *Wolbachia* in insects, has also been demonstrated in the three isopod species *Porcellio dilatatus petiti, Porcellio dilatatus dilatatus*, and *Cylisticus convexus* (Legrand et al., 1978; Moret et al., 2001; Sicard et al., 2014a). *Wolbachia*-induced CI appears when crosses between infected males and uninfected females (or females infected with a different *Wolbachia* strain) are unfertile. As for feminization, the precise mechanism used by *Wolbachia* to induce CI has not yet been unraveled. However, the expected consequence of this phenotype is to reduce gene flow between groups of individuals that differ regarding their *Wolbachia* infection status, possibly playing a role in speciation (Bordenstein et al., 2001; Brucker and Bordenstein, 2012).

Overall, *Wolbachia* have been identified in a large set of terrestrial isopods with an estimated prevalence of about 60%, revealing a pandemic distribution in this group of animals (Bouchon et al., 2008; Cordaux et al., 2012). *Wolbachia* form a monophyletic lineage in which 16 phylogenetic supergroups (from A to Q) have been identified to date (Ramírez-Puebla et al., 2015). Until recently, all isopod-borne *Wolbachia* strains, mostly sampled in temperate regions, belonged to the B supergroup (Cordaux et al., 2004, 2012; Bouchon et al., 2008). However, a recent survey of *Wolbachia* in neotropical isopod species revealed the presence of strains from A and F supergroups (Zimmermann et al., 2015) and we may expect to uncover an even higher *Wolbachia* diversity by screening terrestrial isopods in regions that have not been extensively studied thus far. The pandemic infection of *Wolbachia* in terrestrial isopods might be facilitated by the ability of these bacteria to switch from one host to another (i.e., horizontal transmission). This has been demonstrated in terrestrial isopods where *Wolbachia* transinfections have been achieved through hemolymph contact (Rigaud and Juchault, 1995), cannibalism or predation (Le Clec’h et al., 2013a). Cannibalism is quite common in terrestrial isopods and these investigations demonstrate that *Wolbachia* are capable of crossing the intestine barrier and to survive, at least transiently, in a new host. These observations are congruent with the general view inferred from phylogenetic studies that the wide distribution of *Wolbachia* in terrestrial isopods may result from successful host switching over evolutionary time scales (Bouchon et al., 1998; Cordaux et al., 2001; Zimmermann et al., 2015). Moreover, experimental transinfections have demonstrated that host switching could increase the virulence of *Wolbachia* (Le Clec’h et al., 2012, 2013b).

Is *Wolbachia* only a sex parasite? Due to its endocellular lifestyle, *Wolbachia* has to face the host’s immune defenses (Sicard et al., 2014b). However, live *Wolbachia* have been observed in the hemocytes (immune cells) of *A. vulgare* (Braquart-Varnier et al., 2008; Chevalier et al., 2011). Moreover, hematopoietic organs that produce hemocytes are also infected (Chevalier et al., 2011; Braquart-Varnier et al., 2015b), constituting a niche where *Wolbachia* could potentially initiate and maintain its systemic infection. These intriguing observations raise the question of the efficiency of the host’s immune response. In fact, a negative impact of *Wolbachia* on host immune competence has been demonstrated in *A. vulgare* and *P. dilatatus*, decreasing the survival of infected individuals (Braquart-Varnier et al., 2008; Pigeault et al., 2014). Both cellular (i.e., hemocytes density) and humoral parameters (i.e., phenoloxidase activity) of the host immune response are impacted, leading to septicemia (Sicard et al., 2010). Immune response is also impacted at the molecular level: genes encoding antimicrobial peptides or involved in pathogen recognition, detoxification and autophagy are upregulated in hemocytes of infected individuals, whereas the same gene set is downregulated in ovaries or in whole bodies (Chevalier et al., 2012). These findings underline the fact that each tissue represents a particular niche with specific interactions between the host and *Wolbachia*. Therefore, hosts should be considered as complex ecosystems composed of a mosaic of microhabitats (Dittmer et al., 2014, 2016; Sicard et al., 2014b). Meanwhile, it has been demonstrated that *Wolbachia* can also confer protection against pathogenic bacteria by inducing a better resistance to pathogen infection in *A. vulgare* and *P. dilatatus* (Braquart-Varnier et al., 2015a), similar to *Wolbachia*-mediated protection against viruses and *Plasmodium* parasites in various insects (Hedges et al., 2008; Teixeira et al., 2008; Moreira et al., 2009; Osborne et al., 2009; Hughes et al., 2011; Blagrove et al., 2012; Bian et al., 2013). Taken together, the phenotype of the association between *Wolbachia* and isopods is the result of a subtle balance between detrimental and beneficial effects on host fitness, depending on how we look at the interactions.

### *Rickettsiella*, A Model to Study Transitions between Pathogenicity and Mutualism?

**FIGURE 3:**
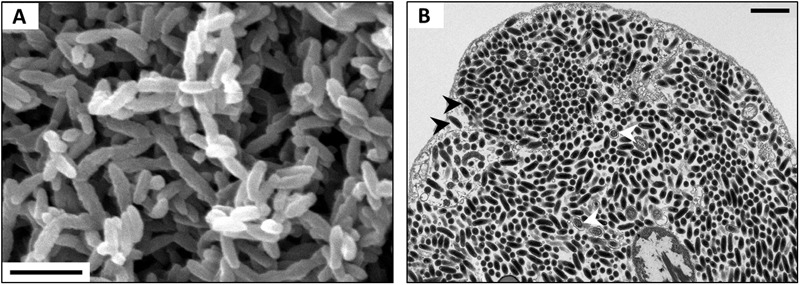
**(A)** SEM of *Rickettsiella* in the hemolymph of *P. scaber*. **(B)** TEM of *Rickettsiella* in an ovary of *Atlantoscia floridana*, showing bacterial cells of two different stages: rod-shaped Elementary Bodies (black arrowheads) and replicative Reticulate Bodies (white arrowheads). Scale bar: 1 μm. Picture credit: Maryline Raimond **(A,B)** and Bianca Lais Zimmermann **(B)**.

Bacteria of the genus *Rickettsiella* are Gammaproteobacteria closely related to *Legionella* and *Coxiella* (Roux et al., 1997; Cordaux et al., 2007; Leclerque, 2008). They are facultative endosymbionts that are mainly known as arthropod pathogens (reviewed in Bouchon et al., 2011). *Rickettsiella*-induced diseases were first reported from the Japanese beetle *Popillia japonica* (Dutky and Gooden, 1952) and since then have been described from numerous insects encompassing lepidopterans, orthopterans, dipterans, dyctiopterans, coleopterans, hymenopterans and hemipterans, as well as arachnids and crustaceans (reviewed in Bouchon et al., 2011). Vago et al. (1970) first described a *Rickettsiella* infection in terrestrial isopods in specimens of *A. vulgare* and therefore proposed the pathotype designation ‘*R. armadillidii.*’ Since then, *Rickettsiella* diseases have been reported in numerous terrestrial isopod species (**Figure 3**), i.e., *A. vulgare, A. nasatum, A. granulatum, Eluma purpurascens, Oniscus asellus, Porcellio laevis*, and *Porcellio gallicus* (Yousfi, 1976), *Helleria brevicornis* and *Philoscia muscorum* (Cordaux et al., 2007); *P. dilatatus* (Federici, 1984), *Porcellio scaber* (Abd and Holdich, 1987; Kleespies et al., 2014) and *Atlantoscia floridana* (**Figure 3B**). *Rickettsiella* have also been detected in the freshwater isopod *Asellus aquaticus* (Wang et al., 2007). As in other arthropod hosts, *Rickettsiella* cause a slowly developing but highly contagious disease in terrestrial isopods, resulting in death of infected individuals (Vago et al., 1970; Bouchon et al., 2011). Diseased individuals are easy to recognize in the later stages of infection due to a white substance in the body cavity that is visible through the ventral body surface.

Historically, there has been a lot of confusion regarding taxonomic diversity and phylogenetic relationships within the genus *Rickettsiella* (Roux et al., 1997; Cordaux et al., 2007; Leclerque, 2008). As such, the entire genus *Rickettsiella* had been initially misassigned to the order Rickettsiales, a group of intracellular Alphaproteobacteria (Weiss et al., 1984), or alternatively to the Chlamydiales (Federici, 1980b). Indeed, microscopic observations of symptomatic specimens from diverse hosts have described *Rickettsiella* replication cycles similar in many aspects to the characteristic cycle of the Chlamydiales. Moreover, *Rickettsiella* species denominations have generally been made based on host species or pathotype, leading to confusing and controversial taxonomic identifications. This is illustrated by the *Rickettsiella* genome sequencing project (Genome Project AAQJ00000000): while this *Rickettsiella* strain had been isolated from a terrestrial isopod, it was named after the cricket pathogen *Rickettsiella grylli*, although these two strains are only distantly related based on molecular markers (Leclerque and Kleespies, 2012; Kleespies et al., 2014). Another case of taxonomic misclassification is exemplified by symbionts of the tick *Ixodes ricinus*, which were initially identified as representatives of a new genus called ‘*Diplorickettsia’* (*Diplorickettsia massiliensis;* Mediannikov et al., 2010), but later included within the genus *Rickettsiella* (Leclerque and Kleespies, 2012; Kleespies et al., 2014). Most importantly, *D. massiliensis* has been identified in three human patients with suspected tick-borne infections, thus establishing its role as a human pathogen (Subramanian et al., 2012). This situation highlights that *Rickettsiella* is still poorly studied and that our knowledge is based on patchy observations across diverse host organisms.

Over the last decade, however, a growing body of molecular data has been obtained from diverse arthropods as well as environmental samples. Based on the current 16S rRNA gene phylogeny, the most widely used marker gene, the *Rickettsiella* genus appears monophyletic but highly diverse, including sub-clades that do not follow the pathotypes nor host origin (Kleespies et al., 2014). These data confirm that *Rickettsiella* are widespread, not only detected in symptomatic hosts but also in healthy individuals and in the environment [e.g., rivers, sea water, and even in aerosol samples (Kleespies et al., 2014)]. In terrestrial isopods, *Rickettsiella* are observed in genetically diverse hosts (Cordaux et al., 2007; Bouchon et al., 2011; Kleespies et al., 2014), reflecting extensive horizontal transfers that might be facilitated by the ability of these bacteria to survive in the soil outside of their hosts for years (Hurpin and Robert, 1976).

The versatility of the *Rickettsiella* is also demonstrated regarding their phenotypes and biological traits. Non-pathogenic *Rickettsiella* strains, phylogenetically distinct from the pathogenic *Rickettsiella*, have recently been reported in the pea aphid *Acyrthosiphon pisum* (Tsuchida et al., 2010, 2014). These *Rickettsiella* are vertically transmitted through transovarial passage, behave as mutualists with no detrimental effects on their hosts and confer protection against predators by changing their host’s body color (Tsuchida et al., 2010, 2014). Vertically transmitted *Rickettsiella* have also been identified in the leafhopper *Orosius albicinctus* (Iasur-Kruh et al., 2013). In the same manner, *Rickettsiella* that are very common and diverse in ticks, are present in eggs laid by infected females, suggesting vertical transmissions (Duron et al., 2016).

In conclusion, the biology of most *Rickettsiella* strains remains to be characterized but our growing knowledge on diversity and phylogeny, although incomplete, suggests that members of the *Rickettsiella* genus use diverse strategies to spread and persist in host populations. Overall, *Rickettsiella* appears as a very promising model to study evolutionary transitions between pathogenicity and mutualism.

### *Rhabdochlamydia porcellionis*, A Model to Study Complex Pathogen Life Cycles

*Rhabdochlamydia porcellionis* are obligate intracellular bacteria so far only observed in the midgut caeca (hepatopancreas) of *P. scaber* (Shay et al., 1985; Drobne et al., 1999; Kostanjsek et al., 2004b). Together with the cockroach pathogen *Rhabdochlamydia crassificans* (Corsaro et al., 2007), *R. porcellionis* represents a new family (the Rhabdochlamydiacaea) within the phylum Chlamydiae (reviewed in Horn, 2008). The Chlamydiae are obligate intracellular pathogens infecting a wide range of Eukaryotes, including protists (amoebae), insects, fishes, mammals, and humans (reviewed in Horn, 2008). They have probably lived in association with eukaryotic hosts for 100s of millions of years and may even have played an essential role in the establishment of plastids in the primary photosynthetic Eukaryote, despite being absent from extant plants (Huang and Gogarten, 2007). The Rhabdochlamydiacaea are most closely related (87% 16S rRNA gene sequence similarity) to the family Simkaniacaea, comprising the two bacteriocyte-associated insect endosymbionts *Candidatus* Fritschea bemisiae from whiteflies and *Candidatus* Fritschea eriococci from scale insects (Thao et al., 2003; Everett et al., 2005; Horn, 2008). In line with this phylogenetic position, *R. porcellionis* can be successfully cultured in insect cell lines, indicating its capacity to infect diverse arthropod hosts (Sixt et al., 2013).

*Rhabdochlamydia porcellionis* exhibits the complex developmental cycle that is characteristic for all Chlamydiae, consisting of replicative reticulate bodies (RBs), intermediate bodies (IBs), and infectious elementary bodies (EBs; Drobne et al., 1999; Kostanjsek et al., 2004b). EBs are rod-shaped cells with a dense cytoplasm and a distinctive, five-layered cell wall. After infection of a host cell, they develop into RBs – large, spherical cells with a three-layered cell wall that replicate within intracellular membrane-bound vacuoles. During the advanced stages of infection, aggregations of vacuoles densely packed with bacteria accumulate in the infected cell, leaving visible signs of infection (white spots) on the surface of the midgut caeca (Drobne et al., 1999; Kostanjsek et al., 2004b). At the end of the infection cycle, RBs redifferentiate into a new generation of infectious elementary bodies and vacuoles are released into the lumen of the hepatopancreas, disrupting the cell membrane in the process (Drobne et al., 1999; Kostanjsek et al., 2004b). Based on this infectious life cycle and the damage caused to the host tissue, the relationship between *R. porcellionis* and its isopod host is clearly parasitic. Moreover, this bacterium has been shown to inhibit apoptosis as a defense mechanism in infected insect cell lines (Sixt et al., 2013). However, many aspects of this symbiosis, including transmission routes and prevalence in other terrestrial isopod species, remain to be elucidated.

### *Hepatincola* and *Hepatoplasma*, Models to Study Nutritional Symbiosis and Symbiont-Symbiont Interactions?

The hepatopancreas (digestive midgut glands) of terrestrial isopods is responsible for the production and secretion of digestive enzymes and involved in the absorption of digestively released nutrients. Moreover, this organ has been demonstrated to harbor dense populations of bacteria in *P. dilatatus* (Donadey and Besse, 1972), *P. scaber* (Wood and Griffiths, 1988; Hames and Hopkin, 1989; Zimmer and Topp, 1998a,b; Zimmer, 1999), *Oniscus asellus* (Wood and Griffiths, 1988; Hames and Hopkin, 1989), and *Ligia pallasii* (Zimmer et al., 2001). Based on feeding experiments and correlations between digestive processes and bacterial densities, hepatopancreatic bacteria have been suggested to be involved in the hydrolysis of cellulose (Zimmer and Topp, 1998a; Zimmer et al., 2002) and the oxidative breakdown of lignins (Zimmer and Topp, 1998b) and tannins (Zimmer, 1999). Therefore, hepatopancreatic symbionts of terrestrial isopods might enable isopods to digest leaf litter and contribute directly to a fundamental ecosystem process, namely litter decomposition. If symbionts facilitate the digestion of leaf litter in terrestrial isopods (Zimmer and Topp, 1998a,b), the existence of hepatopancreatic bacteria might have aided the colonization of terrestrial habitats and thus the evolution of terrestrial isopods through subsequent adaptive radiation. The detritivorous freshwater isopod *A. aquaticus* has been shown to host a relatively diverse hepatopancreatic microbiota (Wang et al., 2007) that seems to contribute to cellulose hydrolysis and phenol oxidation (Zimmer and Bartholmé, 2003). It was only recently that bacterial inhabitants of the hepatopancreas were demonstrated in a marine isopod species, namely *Idotea balthica* feeding on angiosperm tissue in a low-salinity brackish environment (Zimmer et al., 2001, 2002; Wang et al., 2007; Mattila et al., 2014). Elimination of these bacteria through antibiotic treatment did not affect isopod growth on their angiosperm diet (Mattila et al., 2014), questioning a bacterial role in digestive processes (see above).

**FIGURE 4:**
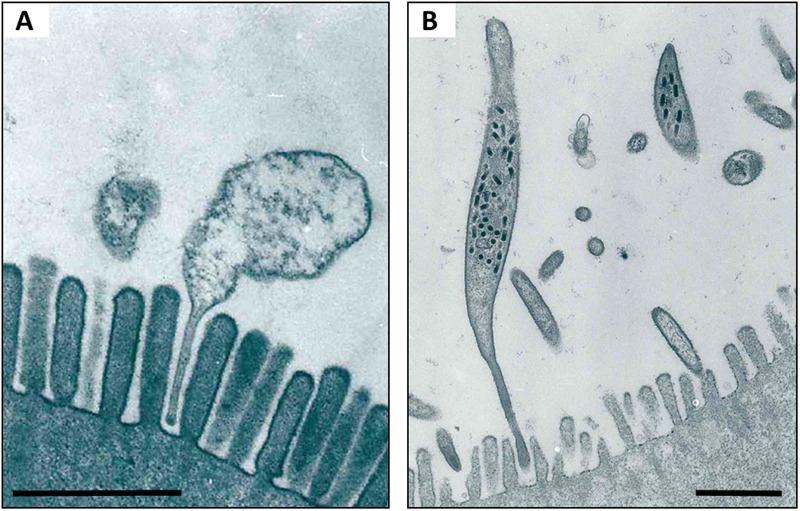
**TEM of *Hepatoplasma***(A)** and *Hepatincola***(B)** in the lumen of the hepatopancreas (midgut glands) of *P. scaber*.** Note how both bacteria attach to the microvillous border of the host cell via stalk-like appendages. Scale bars: 1 μm. Picture credit: Yongjie Wang.

Two of these hepatopancreatic symbionts that occur extracellularly in the lumen and attach to the epithelial microvillous brush border of the cells via stalk-like appendages (**Figure 4**) were identified based on 16S rRNA gene sequences: *Candidatus* Hepatoplasma crinochetorum (Mollicutes, hereafter ‘*Hepatoplasma*’; Wang et al., 2004a) and *Candidatus* Hepatincola porcellionum (Rickettsiales, hereafter ‘*Hepatincola*’; Wang et al., 2004b). Since then, *Hepatoplasma* has been detected in all Oniscidean species tested for hepatopancreatic bacteria (except *Ligidium hypnorum*), whereas *Hepatincola* has only been observed in species of the Crinocheta (the most terrestrial group; Fraune and Zimmer, 2008). No individual specimen was found to host both symbionts simultaneously (Wang et al., 2007; Fraune and Zimmer, 2008), suggesting some, albeit hitherto unknown, mechanism of mutual exclusion. The intertidal species *Ligia oceanica* can harbor either *Hepatoplasma* or *Pseudomonas* sp. in their hepatopancreas (Wang et al., 2007; Fraune and Zimmer, 2008). Similarly, *Hepatoplasma* (but not *Hepatincola*) has been observed in two other intertidal species, *Ligia occidentalis* and *L. pallasii* (Eberl, 2010). The evolutionary or phylogenetic relevance of this finding remains unclear. According to highly congruent 16S rRNA- and 18S rRNA-based phylogenetic trees of *Hepatoplasma* from different isopod species and their isopod hosts, respectively, host–symbiont specificity and co-evolution with little horizontal exchange among host species has been assumed (Fraune and Zimmer, 2008). Taking into account that *Hepatoplasma* increases its host’s survival on a cellulosic low-quality diet (Fraune and Zimmer, 2008), the acquisition of this symbiont at an early stage of the evolution of terrestrial isopods may be considered a predisposition for the successful colonization of diverse land habitats and the use of terrestrial food sources. In *Ligia oceanica*, a representative of a prototypal isopod genus, gene sequences that resemble known carbohydrases of the GH45 family could be isolated from individuals harboring hepatopancreatic symbionts, but not from aposymbiotic individuals or from *P. scaber* (S. Fraune and M. Zimmer, personal communication). The latter species was later shown to possess an endogenous cellulase gene belonging to the GH9 family (Kostanjsek et al., 2010). The recently published genome of *Hepatoplasma* from *A. vulgare* (Leclercq et al., 2014) allows the identification of candidate genes that are potentially involved in cellulolytic processes: five coding sequences were assigned to enzyme classes defined by the Carbohydrate-Active Enzyme Database CAZy (Cantarel et al., 2009; Lombard et al., 2014), including the glycoside hydrolase family GH13, two glycosyltransferases and two carbohydrate esterases (**Table 1**; J. Dittmer, Ph.D. thesis; D. Bouchon, personal communication). However, the transcriptome of *A. vulgare* (Chevalier et al., 2012) also contains a set of genes of potentially cellulolytic enzymes (J. Dittmer, Ph.D. thesis; D. Bouchon, personal communication), including several orthologs of the endogenous cellulase gene of *P. scaber* (Kostanjsek et al., 2010). Functional analyses of these candidate genes will determine whether the cellulolytic genes of *Hepatoplasma* are partly redundant or instead complement endogenous host genes. Despite the fact that *P. scaber* possesses an endogenous cellulase gene (Kostanjsek et al., 2010), the cellulase activity inside its hindgut lumen correlates with the number of bacterial cells in the hepatopancreas (Zimmer and Topp, 1998a).

**Table 1:** Carbohydrate-active enzymes in the genome of *Hepatoplasma crinochetorum* based on the CAZy database (Cantarel et al., 2009; Lombard et al., 2014).

Enzyme class	Hits	Family	Product
Glycoside hydrolase	1	GH13	Trehalose-6-phosphate hydrolase (EC 3.2.1.93)
Glycosyltransferase	2	GT2	NA
		GT32	Mannosyltransferase OCH1 (EC 2.4.1.232)
Carbohydrate esterase	2	CE9	*N*-acetylglucosamine -6-phosphate deacetylase (EC 3.5.1.25)

In contrast to *Hepatoplasma, Hepatincola* reduces its host’s longevity (Fraune and Zimmer, 2008). The relationship between *Hepatincola* and its isopod hosts is evolutionarily much younger than that of the more ancient *Hepatoplasma* (Wang et al., 2007). The presence of stalk-like cytoplasmic appendages that both *Hepatoplasma* and *Hepatincola* insert into the epithelial brush border of hepatopancreatic cells (**Figure 4**) has prompted the hypothesis that the evolutionarily young parasite (*Hepatincola*) invaded the evolutionarily ancient mutualistic relationship of its host with *Hepatoplasma* through molecular mimicry (Fraune and Zimmer, 2008). As mentioned above, the two symbionts seem to mutually exclude each other, and it is tempting to speculate that the presence of the mutualist prevents the parasite from colonizing the hepatopancreas.

Contradictory to the hypothesis that *Hepatoplasma* might have facilitated the success of isopods during the early stages of terrestrialization, this symbiont exhibits low prevalence in some populations (Wang et al., 2007). This is potentially due to environmental transmission rather than vertical transmission from mother to offspring (Wang et al., 2007). Hence, *Hepatoplasma* cells appear to be ingested along with inoculated food sources, through coprophagy or through cannibalism (Brandstädter and Zimmer, 2008). These observations suggest that *Hepatoplasma* might have become less relevant or redundant, potentially upon the evolutionary acquisition of endogenous cellulases by isopods (see above). Alternatively, this particular symbiosis may not have been significant for the evolutionary success of isopods on land because of its contribution to digestive processes but, if any, because of other beneficial roles, such as preventing pathogenic or parasitic infections.

From an evolutionary point of view, it is interesting to consider the presence of hepatopancreatic bacteria in thus far only one of the screened marine isopod species, and only in a population from a low-salinity brackish environment (Mattila et al., 2014). This observation is in line with Plante et al. (1990), reasoning that the conditions within marine invertebrate guts differ less from the external microbial environment than that in terrestrial or freshwater species, rendering it not necessarily advantageous for a marine bacterium to colonize the gut of an invertebrate instead of living in seawater. Further detailed studies on the suitability of marine and brackish isopod species as hosts for bacterial symbionts are warranted to better understand the evolutionary and ecological background of the symbiotic association of isopods and intestinal symbionts.

### *Bacilloplasma*, A Model to Study Ancient Host–Symbiont Relationships?

*Candidatus* Bacilloplasma (hereafter ‘*Bacilloplasma*’) represents a lineage of Mollicutes (phylum Tenericutes) associated with the hindgut cuticle of terrestrial isopods (Kostanjsek et al., 2007). It was first described in *P. scaber* and *Ligidium hypnorum* (Drobne, 1995; Kostanjsek et al., 2007) and recently it was also detected in the hindgut of *A. vulgare* using pyrotag sequencing (Dittmer et al., 2016). *Bacilloplasma* are wall-less, rod-shaped extracellular bacteria that colonize the hindgut surface in *P. scaber* via attachment to the cuticular spines protruding into the gut lumen, suggesting a highly specific adaptation to the isopod gut environment (Kostanjsek et al., 2007). While their precise role in the terrestrial isopod hindgut remains unknown, a non-pathogenic commensal relationship seems likely due to the absence of disease symptoms in infected individuals (Kostanjsek et al., 2007). Nonetheless, a detailed investigation of isopod fitness in relation with *Bacilloplasma* infection is still needed. Likewise, nothing is known regarding symbiont acquisition and transmission. Interestingly, the hindgut cuticle is regularly shed during molting, raising the question how the infection is maintained or reestablished after this disturbance. A possible mechanism might be to reingest the shed cuticle, which is common in terrestrial isopods and assumed to restore minerals lost during the molt (Kostanjsek et al., 2007). Similarly, cannibalism may also allow for horizontal transmission between individuals, as *Bacilloplasma* has never been detected in soil or associated with plant litter or isopod feces.

The bacterium has not been cultured and its 16S rRNA gene has less than 83% sequence similarity with other Mollicutes clades (Kostanjsek et al., 2007). *Candidatus* Lumbricincola, a gut symbiont of earthworms, is distantly related to *Bacilloplasma* based on the 16S rRNA gene (Nechitaylo et al., 2009). Interestingly, isopods and earthworms occupy similar ecological niches within the soil macrofauna. Therefore, it would be highly relevant to obtain more molecular data allowing us to potentially discover past symbiont transfers between the two hosts. Moreover, several close relatives of *Bacilloplasma* have recently been observed in the mid- and hindguts of several marine decapod crustaceans, namely the shrimps *Rimicaris exoculata* (Durand et al., 2010) and *Litopenaeus vannamei* (Zhang et al., 2014), as well as the Chinese Mitten Crab *Eriocheir sinensis* (Chen et al., 2015; Zhang et al., 2016). In the latter case, symbionts closely related to both *Bacilloplasma* and *Lumbricincola* even represent the dominant taxa in the gut, but are absent from gills and the surrounding water, suggesting that these bacteria are specialized resident gut symbionts (Zhang et al., 2016). Moreover, microscopic observations of the Mitten Crab hindgut revealed highly abundant, rod-shaped bacteria attached directly to the epithelium surface (instead of the cuticular spines) using pili-like structures for adherence (Chen et al., 2015). While it is tempting to speculate that *Bacilloplasma* has evolved from a marine ancestor still common in aquatic crustaceans today, and has therefore maintained a long-lasting symbiotic association with terrestrial isopods, more research is clearly needed in order to better understand this relationship.

### Viruses and Acanthocephalan Parasites, Models to Study Host Manipulations by Non-bacterial Symbionts

Animal-associated microbiotas are not limited to bacterial symbionts but can also include other microorganisms, such as viruses, archaea, protists or Eukaryotic parasites, which may have equally strong impacts on host fitness as the bacterial members of the microbiota. A classic example for this is the diverse microbial community in the hindgut of lower termites, consisting of bacteria, archaea and protists, all of which are involved in digestive processes (Hongoh, 2010; He et al., 2013; Peterson et al., 2015). Although our knowledge regarding non-bacterial microbes in terrestrial isopods is still very limited, two other microorganisms are known to manipulate their isopod hosts in various ways: an Invertebrate Iridescent Virus (IIV) and the acantocephalan parasite *Plagiorhynchus cylindraceus*.

Invertebrate Iridescent Viruses (genus: *Iridovirus*, family: Iridoviridae) are DNA viruses known to infect diverse invertebrates, mainly terrestrial isopods and dipteran insects, but also molluscs, annelids and nematodes (Williams, 2008). They replicate in the cytoplasm of infected cells and form large, icosahedral virions arranged into paracrystalline arrays (Lupetti et al., 2013). Their characteristic feature is the iridescent color caused by light reflection from the paracrystalline virion arrays in infected tissues. As such, iridovirus infection in terrestrial isopods is manifested by a change in body color, as infected individuals turn purple-blue (Cole and Morris, 1980; Federici, 1980a; Lupetti et al., 2013). This discoloration starts as a blue bloom on the unpigmented ventral side of the animal, which then migrates to the pigmented dorsal cuticle, before the entire body turns a completely iridescent blue (Wijnhoven and Berg, 1999; Lupetti et al., 2013). Infection is also accompanied by behavioral changes in that heavily infected individuals show a decreased response to phototactic stimuli or water contact and produce less feces, indicative of decreased food consumption (Wijnhoven and Berg, 1999; Lupetti et al., 2013). Virion aggregates have been observed in various tissues, such as epidermal, muscle and fat body cells, the hindgut, reproductive tissues, nerve cells and hemocytes (Cole and Morris, 1980; Federici, 1980a; Lupetti et al., 2013). Diseased cells are generally hypertrophied due to viral replication and the accumulation of large numbers of virions in the cytoplasm and infected specimens generally die within 30 days post-infection (Federici, 1980a; Lupetti et al., 2013).

The iridovirus was first isolated from *A. vulgare, P. scaber* and *P. dilatatus*, and was initially named Isopod Iridescent Viruses, a term that was later generalized to IIV (Cole and Morris, 1980; Federici, 1980a). Since then, iridovirus infection has been observed in 25 terrestrial isopod species from all over the world (Wijnhoven and Berg, 1999; Williams, 2008; Karasawa et al., 2012; Lupetti et al., 2013). Although the mechanism of infection in the wild is not completely understood, it has been hypothesized that infections can be acquired via wound contact, cannibalism on diseased conspecifics or corpophagy (Cole and Morris, 1980).

Apart from this particular virus, a recent study in *A. vulgare* indicates that the diversity of viruses in terrestrial isopods may be far greater than anticipated. Indeed, a genome-wide survey revealed no less than 54 endogenous viral elements (EVEs), representing 10 viral lineages from five viral families, integrated in the genome of *A. vulgare* (Theze et al., 2014). Moreover, many of these endogenized virus particles have uninterrupted Open Reading Frames, indicating the recent endogenization of viruses probably currently present in isopod populations (Theze et al., 2014).

Besides viruses, infection with Eukaryotic parasites can have far-reaching consequences for the host organism. Indeed, many parasites spend parts of their life cycle in an intermediate host that eventually needs to be consumed by a definitive host, in which the parasite can then reach maturity. Consequently, parasites have evolved elaborate strategies to manipulate the behavior of their intermediate host in that it facilitates encounters with potential definitive hosts. An example for such a host manipulation can be observed in terrestrial isopods infected with the acanthocephalan *P. cylindraceus*, a parasite of birds that uses terrestrial isopods as intermediate hosts (Schmidt and Olsen, 1964). During the parasite’s life cycle, eggs are excreted with the bird’s feces and ingested by terrestrial isopods. The acanthor hatches within the isopod gut, penetrates the gut epithelium and remains there in a state of dormancy for up to 25 days. Then, it migrates through the gut wall into the body cavity, where it develops into an infective cystacanth within 60–65 days post-infection. When an infected isopod is ingested by a bird, the parasite’s definitive host, *P. cylindraceus* attaches itself to the bird’s gut wall and develops into a mature worm (Schmidt and Olsen, 1964).

*Plagiorhynchus cylindraceus* has been detected in natural populations of six isopod species, namely *A. vulgare, A. versicolour, Hemilepistus fedtschenkoi, H. reductus, P. scaber*, and *Trachelipus squamuliger* (Moore, 1983; Levri and Coppola, 2004; Dimitrova, 2009). Although parasite prevalence and infection intensity are generally very low, *P. cylindraceus* infection has significant effects on their isopod host. First, acanthocephalan infection alters isopod behavior in that infected individuals spend more time in dry, exposed and unsheltered areas, making them easier to detect by bird predators than their uninfected conspecifics (Moore,. 1983). Second, the parasite reduces female reproductive potential, as ovaries of infected females often fail to develop normally, while the testes of infected males appear unaffected (Schmidt and Olsen, 1964; Moore, 1983; Dimitrova, 2009).

*Plagiorhynchus cylindraceus* infection generally triggers an immune response by the isopod host, manifested by hemocyte aggregations and melanisation of the parasite at the site of penetration into the gut epithelium (Schmidt and Olsen, 1964; Dappen and Nickol, 1981). However, resistance against *P. cylindraceus* is age-dependent, as more than 90% of adult *A. vulgare* (more than 1 year old) successfully encapsulated and eliminated the intruder in laboratory experiments, while more than 70% of isopods under 9 months of age failed to mount an immune response and became infected (Nickol and Dappen, 1982). This high level of resistance observed in adult isopods may explain why parasite prevalence is generally very low in natural populations.

## The Big Picture: The Terrestrial Isopod Microbiome

### Bacterial Diversity – There is More Than Meets the Eye

**FIGURE 5:**
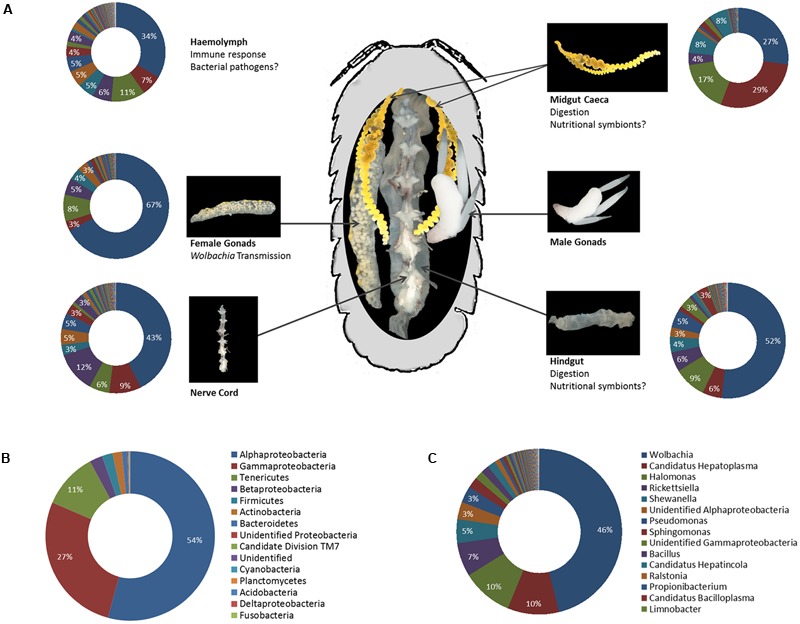
**(A)** Representation of the microbiota in different tissues of *A. vulgare* (modified from Dittmer et al., 2016). Note that data from males and females are combined, therefore the chart illustrating the microbiota in the gonads represents both male and female gonads. The legend for all charts is the same as in **(C)**. **(B,C)** The microbiome of *A. vulgare* for all tissues combined, at the phylum **(B)** and genus **(C)** level. Proteobacteria are represented at class level. Legends show the 15 most abundant phyla/classes and genera, respectively.

Apart from the bacterial endosymbionts described above, all of which are specifically adapted to the within-host or even intracellular environment, the role of more diverse microbial communities in terrestrial isopod ecology has been investigated for several decades (reviewed in Zimmer, 2006). Early molecular studies of isopod microbiota focussed exclusively on digestive tissues, due to the interest in bacteria potentially involved in nutritional processes. Despite being based only on relatively small 16S rRNA gene clone libraries, this work already hinted at a high bacterial diversity: numerous taxa from the phyla Bacteroidetes, Firmicutes, and Proteobacteria (e.g., *Bacillus, Bacteroides, Enterococcus, Pseudomonas*) have been observed in the hindgut of *P. scaber* (Kostanjsek et al., 2002, 2004a; Lapanje et al., 2010) and diverse Proteobacteria (*Aeromonas, Burkholderia, Ralstonia, Rhodobacter, Vibrio*) in the midgut caeca of the freshwater isopod *A. aquaticus* (Wang et al., 2007) and the semiterrestrial species *L. pallasii* and *Ligia occidentalis* (Eberl, 2010). A recent in-depth pyrotag screening of the bacterial community in several tissues of *A. vulgare* from laboratory-reared lineages as well as populations sampled in the field (Dittmer et al., 2016) provides us with the most detailed picture of the isopod microbiome to date. In total, 208 bacterial genera from 31 classes and 19 phyla were observed in this study (Supplementary Table S1), revealing a high bacterial diversity not just in the gut, but in all tested tissues, including midgut caeca, gonads, nerve cord and hemolymph (**Figure 5A**). 28 additional genera were detected in the feces of the same individuals but were absent from the tissues. Alphaproteobacteria, Gammaproteobacteria, and Mollicutes together represented 92% of all sequences due to highly predominant taxa (i.e., *Wolbachia, Halomonas, Pseudomonas, Rickettsiella, Shewanella*, and *Hepatoplasma*), while Firmicutes, Actinobacteria, and Bacteroidetes represented 2, 1.8, and 1% of all sequences, respectively (**Figures 5B,C**). However, the phylum Actinobacteria was the second most represented phylum in terms of taxonomic richness, with 54 observed genera, after the Proteobacteria with 88 genera (Supplementary Table S1).

Another recently published investigation of the hindgut microbiome in *P. scaber* using Illumina sequencing (Horvathova et al., 2016) enables us for the first time to compare the gut microbiomes of two isopod species: while the gut microbiome of *P. scaber* was similar to that of *A. vulgare* in terms of bacterial diversity at the phylum level (20 phyla), microbiome composition was quite different: Proteobacteria represented by far the most abundant phylum in both species (approximately 85%) but Alphaproteobacteria were more abundant than Gammaproteobacteria in *A. vulgare*, whereas the opposite was the case in *P. scaber*. Similarly, the Mollicutes *Hepatoplasma* and *Bacilloplasma* accounted for 8.5% of the gut microbiome in *A. vulgare*, while they were negligible in *P. scaber* (0.72%). In contrast, Bacteroidetes and Actinobacteria were more highly represented in *P. scaber* than in *A. vulgare*.

While more microbiomes from ecologically diverse species are clearly needed in order to establish potential species-specific patterns, these two studies already demonstrate that the terrestrial isopod microbiome can be highly diverse and contain 100s of taxa, even in the presence of highly predominant endosymbionts such as *Wolbachia* or *Hepatoplasma*. Investigating how these two fractions of the microbiome (i.e., highly abundant heritable endosymbionts and the rare bacterial biosphere) interact, either directly or indirectly (e.g., via regulation of the host immune response), represents an interesting path for future research.

Another intriguing aspect is the fact that all major tissues were found to harbor a high bacterial diversity in *A. vulgare* (Dittmer et al., 2016). While it is obvious that some bacteria still have their preferred niches (e.g., *Hepatoplasma* is most abundant in the caeca although it can be present in other tissues as well), it seems that isopods are not only fairly permeable to bacteria but that bacteria can also spread easily from one tissue to another. This is in line with previous findings regarding *Wolbachia* tissue tropisms in isopods, since (i) *Wolbachia* is able to cross the gut barrier and infect several other tissues after transfer via cannibalism (Le Clec’h et al., 2013a) and (ii) the cyclically produced oocytes are probably infected with *Wolbachia* from somatic tissues (Genty et al., 2014). Circulating hemocytes most likely serve as vehicles for *Wolbachia* between tissues (Braquart-Varnier et al., 2015b). If such inter-tissue transfers are possible for *Wolbachia*, it is conceivable that other bacteria might be able to adopt similar strategies. In this context, it is of interest that the highest species richness in *A. vulgare* was observed in the hemolymph (Dittmer et al., 2016). While the precise within-host trajectories of isopod-associated bacteria are far from being understood, it is likely that many of them represent commensal or opportunistic bacteria able to occupy various niches in the host, depending on available nutrients or host immunotolerance. From a technical perspective, the possibility to study host–microbiota dynamics at the tissue level due to the large body size of the host nicely illustrates one of the advantages of isopods as a model system.

### Environmental Bacteria – Transient Passengers, Food or Collaborators?

A major finding from the microbiome of *A. vulgare* is that an important fraction of the bacterial community is most likely acquired from environmental sources, since about 70% of all taxa were also detected in feces and/or soil (Dittmer et al., 2016). Although these taxa represent rare members of the microbiome, they may constitute a source of variability between individuals and populations, depending on the microbes prevailing in the environment (e.g., soil or plant food sources). Indeed, different populations of *A. vulgare* harbored different bacterial communities, while the microbiomes of several genetically diverse laboratory-reared lineages were more similar to each other (Dittmer et al., 2014, 2016). The latter excludes different host genotypes as the primary driver of microbiome composition in this species and makes a shift due to environmentally acquired bacteria all the more likely. The effect of different food sources on the gut microbiome has been more formally tested in *P. scaber*, confirming that different food sources shape taxonomic composition of the gut microbiome at the phylum level (Horvathova et al., 2016).

The importance of environmental bacteria for terrestrial isopod health and fitness has attracted considerable attention long before the recent interest in animal microbiomes. Hence, it has been demonstrated that microbial colonization of leaf litter and artificial diets is beneficial for *P. scaber*, as it improves survival and growth rates of juveniles and adults as well as female reproductive success, especially when feeding on low-quality food sources (Zimmer and Topp, 1997, 2000; Kautz et al., 2002; Horvathova et al., 2015, 2016). However, it has proven challenging to elucidate the precise role of the bacteria in causing this positive effect. Several hypotheses have been put forth: (i) Environmental microbes might themselves represent an easily digestible supplementary food source, providing essential or limiting nutrients (Gunnarsson and Tunlid, 1986; Zimmer and Topp, 1998a; Ihnen and Zimmer, 2008). In line with this hypothesis, *P. scaber* has been shown to selectively consume and digest Actinomycetes over other bacteria and fungi, independent of their cellulolytic capacity (Ihnen and Zimmer, 2008). Moreover, recent experiments indicate that microbes acquired from leaf litter or feces may enhance juvenile growth via the provisioning of fatty acids and vitamins (Horvathova et al., 2015). (ii) Microbial enzymes contribute to digestion by preconditioning the food prior to ingestion (“external rumen”) or even through continued enzymatic activity after ingestion (Hassall and Jennings, 1975). Although a direct link between bacterial enzymes and isopod digestion has not been demonstrated to date, the isopod gut contains high amounts of cellulolytic enzymes which most animals cannot produce (Zimmer and Topp, 1998a,b). Nonetheless, there is evidence that the marine isopod *Limnoria quadripunctata* produces numerous endogenous glycosyl hydrolases (King et al., 2010) and two functional cellulases of host origin have been extracted from the midgut caeca in *P. scaber* (Kostanjsek et al., 2010). However, the presence of endogenous cellulolytic enzymes in terrestrial isopods remains to be investigated in more detail in order to determine whether they could solely account for cellulose digestion by isopods or act in synergy with microbial enzymes produced by symbiotic and/or environmental bacteria. (iii) Microbial colonization acts as an indicator of high-quality food, thereby stimulating consumption (Zimmer et al., 2003). This may be due to microbial activity enhancing palatability and nutritive quality of the leaf litter (e.g., by decreasing the C:N ratio and the content of phenolic compounds) prior to ingestion by isopods (see ii). The truth is most likely a combination of all of these and may depend to some extend on the isopod species and its feeding preferences, the nutritive quality of the food source and the different microbes involved.

## Perspective: Relevance of the Microbiome at the Ecosystem Level?

Considering that researchers are now beginning to unravel the true complexity of the terrestrial isopod microbiome, a challenging objective for future research will be to better understand the functional roles of different bacteria in a wider range of isopod species as well as their interaction with the host organism, other members of the symbiotic community and the environment. Fascinating questions that need to be addressed concern the acquisition of different microbes by juveniles – possible transmission pathways include vertical transmission (either via the egg or the marsupial fluid in the brood pouch), horizontal transmission (via contact with conspecifics, coprophagy, or cannibalism) and environmental transmission (from soil or plant food sources). How plastic or variable is the microbiome throughout an individual’s lifespan – do some microbes establish more stable associations (i.e., become residents) throughout the life cycle while others remain transient? Is there a regular turn-over due to molting? Are environmental microbes just randomly acquired from the environment or is there some control or selection by the host over both microbiome composition and microbial density? Most importantly, does the plasticity of the microbiome represent an adaptive advantage? For instance, do population-specific microbiomes provide the host with microbial functions that are best adapted to the prevailing environmental conditions? These may include certain types of food sources or abiotic environmental factors. As such, it has been demonstrated that mercury pollution in the environment induced a shift in the gut microbiome of *P. scaber* along with an enrichment of mercury-resistant bacteria, which could potentially influence mercury tolerance of the host (Lapanje et al., 2010). Along the same lines, the microbiome may be essential in mediating the functional role of terrestrial isopods as key decomposers of organic matter and regulators of nutrient cycling in soil ecosystems. As such, isopod grazing on fungi has a stabilizing effect on microbe-mediated nutrient cycling in soils that is predicted to remain stable even under global climate change scenarios (Crowther et al., 2015). Therefore, if changes in the microbiome translate into functional differences for the host (e.g., in terms of health, nutrition, and reproduction), they might ultimately interfere with the regulatory function of isopods in terrestrial ecosystems. If this were the case, a better understanding of the complex interplay between isopods and their microbiome could help predict the stability of ecosystem functions under different ecologically relevant conditions, such as global climate change.

## Concluding Remarks

This review synthesizes our current knowledge on bacterial endosymbionts as well as the microbiome as a whole in terrestrial isopods. We highlight that terrestrial isopods represent an excellent model system to study diverse symbiotic interactions along the spectrum from parasitism to mutualism. Moreover, this group of animals inhabits virtually all types of terrestrial environments from the marine littoral zone to deserts, thereby allowing the study of host–symbiont interactions across large-scale eco-evolutionary gradients. While many aspects remain to be investigated (e.g., the functional roles of particular bacteria in diverse host species, different environmental conditions or in the transition from marine to terrestrial habitats), the meta-omics era now provides us with the necessary toolkit to address such questions even in non-model organisms.

## Data Accessibility

The 16S rRNA gene sequences from the microbiota of *Armadillidium vulgare* have been deposited in the European Nucleotide Archive under the study accession number PRJEB8160.

## Author Contributions

DB, MZ, and JD contributed ideas and figures and wrote the manuscript.

## Conflict of Interest Statement

The authors declare that the research was conducted in the absence of any commercial or financial relationships that could be construed as a potential conflict of interest.
